# Myocardial Work Analysis in ST-Elevation Myocardial Infarction: Insights into Left Ventricular Ejection Fraction—A Pilot Study

**DOI:** 10.3390/life15030338

**Published:** 2025-02-21

**Authors:** Alexandra-Cătălina Frișan, Mihai-Andrei Lazăr, Raluca Șoșdean, Marius Simonescu, Daniel-Miron Brie, Aniko Mornoș, Silvia Ana Luca, Ioana Ionac, Cristian Mornoș

**Affiliations:** 1 Cardiology Department, “Victor Babes” University of Medicine and Pharmacy, 2 Eftimie Murgu Square, 300041 Timisoara, Romania; alexandra.frisan@umft.ro (A.-C.F.); lazar.mihai@umft.ro (M.-A.L.); marius.simonescu@umft.ro (M.S.); silvia.luca@umft.ro (S.A.L.); ionac.ioana@umft.ro (I.I.); mornos.cristian@umft.ro (C.M.); 2 Institute of Cardiovascular Diseases Timisoara, 13A Gheorghe Adam Street, 300310 Timisoara, Romania; brie_daniel@yahoo.com (D.-M.B.); mornosaniko@gmail.com (A.M.); 3 Research Center of the Institute of Cardiovascular Diseases Timisoara, 13A Gheorghe Adam Street, 300310 Timisoara, Romania

**Keywords:** myocardial work, speckle tracking echocardiography, global longitudinal strain, ST-elevation myocardial infarction, adverse outcomes, left ventricular ejection fraction, percutaneous coronary intervention, heart failure, prognosis

## Abstract

(1) Background: Left ventricular ejection fraction (LVEF) is traditionally used to assess prognosis in acute ST-elevation myocardial infarction (STEMI) patients. However, LV myocardial work (MW), evaluated echocardiographically, offers additional prognostic information by considering loading conditions. (2) Methods: This prospective study investigated the prognostic value of MW indices in 119 consecutive STEMI patients treated with primary percutaneous coronary angioplasty, stratified into three LVEF categories: reduced (≤40%), mildly reduced (41–49%), and preserved LVEF (≥50%). Transthoracic echocardiography was performed before discharge, and the primary endpoint included heart failure hospitalization, ventricular arrhythmias, all-cause mortality and new acute coronary syndromes. (3) Results: Patients with preserved or mildly reduced LVEF had higher global longitudinal strain, global work index, global constructive work (GCW), and global work efficiency, as well as lower global wasted work (GWW), compared to those with reduced LVEF. GCW was the strongest predictor of adverse outcomes in the preserved LVEF group (AUC = 0.730, *p* = 0.035), while GWW demonstrated robust predictive performance in the reduced LVEF group (AUC = 0.787, *p* = 0.001). (4) Conclusions: MW indices, particularly GCW and GWW, provide significant prognostic value in distinct LVEF categories in STEMI patients. These findings indicate that MW enhances risk stratification and informs management in this patient population.

## 1. Introduction

In the initial days to months following acute myocardial infarction, left ventricular (LV) dysfunction is a key marker for identifying patients at higher risk of worse outcomes. Advancements in primary percutaneous coronary intervention (PCI) have greatly improved outcomes by restoring blood flow and reducing infarct size. Nevertheless, despite improved revascularization techniques, myocardial injury and adverse remodeling can still occur. The left ventricular ejection fraction (LVEF) has an important prognostic value, reflecting the extent of myocardial damage and functional impairment post-myocardial infarction. It continues to be the most widely used parameter for risk stratification and ongoing monitoring in this clinical setting [1,2,3]. However, LVEF, as a measure of systolic function, has several well-known limitations, including load dependency, significant intraobserver and interobserver variability and insensitivity to subtle reductions in contractility [4,5]. Global longitudinal strain (GLS), derived from speckle tracking echocardiography, is a relatively novel technique that detects subtle systolic dysfunction, even when LVEF appears normal. However, GLS is also load-dependent [6,7]. A newer imaging technique, myocardial work (MW), integrates afterload (estimated by systolic brachial artery pressure) and myocardial deformation (strain) to assess LV function [8,9]. This method, due to its load independence, has demonstrated superior prognostic and diagnostic value compared to LVEF and GLS in patients with coronary artery disease and heart failure [10,11]. We hypothesize that myocardial work indices, by integrating pressure-strain loops, provide superior prognostic information compared to GLS and LVEF, particularly in identifying subclinical myocardial dysfunction and predicting adverse outcomes in acute ST-segment elevation myocardial infarction (STEMI) patients.

The present pilot study aims to evaluate the prognostic value of MW parameters in patients following STEMI, with a particular focus on their predictive capacity across different LVEF categories.

## 2. Materials and Methods

### 2.1. Study Population

This prospective, single-center cohort study included 119 consecutive patients who were admitted to our clinic with a STEMI diagnosis [12], during a period of one year. All patients were successfully treated with primary PCI and underwent a complete echocardiographic examination prior to discharge. Patients were treated according to current STEMI guidelines [13] and followed up for at least six months. Heart failure hospitalization, ventricular arrhythmias, all-cause mortality, and new acute coronary syndromes represented the primary endpoints.

Exclusion criteria included significant variability in R-R intervals (atrial fibrillation, frequent supraventricular or ventricular ectopic beats), paced rhythm, poor image quality (that interfered with the echocardiographic assessment required for speckle tracking analysis), severe valvular disease, and other conditions resulting in a life expectancy of less than 1 year.

Patients were categorized into three groups, based on LVEF at inclusion in the study, according to ESC guidelines [14]: reduced (≤40%), mildly reduced (41–49%) and preserved LVEF (≥50%). Clinical and biological data at the time of admission were obtained from medical records.

The study conforms to the Declaration of Helsinki guidelines and received approval from the Institution’s Scientific Research Ethics Committee (07/31.01.2024). All participants provided informed consent.

### 2.2. Transthoracic Echocardiographic Evaluation

A thorough transthoracic echocardiographic examination, using a Vivid 9 ultrasound system (General Electric Vingmed Ultrasound, Milwaukee, WI, USA), with an M5S transducer, was performed prior to discharge. All images were digitally stored and later analyzed with EchoPac software (version 203, General Electric Vingmed Ultrasound, Horten, Norway). Echocardiographic image acquisition was performed while the patient was in the left lateral decubitus position, synchronized with the electrocardiogram. Blood pressure was measured beforehand using a sphygmomanometer to non-invasively estimate the peak LV pressure. LVEF was measured using the biplane Simpson’s method. Apical four-chamber, two-chamber and long-axis views were obtained at a frame rate exceeding 40 frames per second, covering at least three consecutive cardiac cycles. LV GLS was automatically calculated by identifying the region of interest, with the operator reviewing and adjusting the contours to ensure precise delineation of the LV wall. The final LV GLS value was determined by averaging the peak systolic strain across the three apical views using a 17-segment model.

The Tissue Doppler Imaging (TDI) program was operated in pulsed-wave Doppler mode, with mitral annular motion assessed in the apical four-chamber view at a frame rate of 80–140 frames per second. A sample volume was placed sequentially at the septal and lateral corners of the mitral annulus. The peak early (e’) and late (a’) diastolic velocities were recorded, while the peak systolic velocity (s’) was identified as the maximum velocity during systole, excluding the isovolumic contraction phase. Velocities were recorded during five consecutive cardiac cycles at end-expiratory apnea, and the results were averaged. All TDI signals were acquired with a horizontal time sweep set to 100 mm/s. The mean velocities from the lateral and septal mitral annular sites were calculated.

Standard echocardiographic measurements were assessed in accordance with the guidelines of the European Association of Cardiovascular Imaging and American Society of Echocardiography [15].

### 2.3. Myocardial Work Assessment

MW parameters were calculated using EchoPac software. The non-invasive method for assessing MW had been previously validated and described in the literature [7,8,16,17,18,19]. Pulsed-wave Doppler recordings of mitral and aortic valve inflow signals were used to determine the timing of mitral and aortic valve opening and closure. By integrating LV GLS with systolic blood pressure, the software generated an LV pressure-strain loop. This allowed for the calculation of MW indices:

- Global work index (GWI): Represents the total myocardial work performed from mitral valve closure to its opening, reflecting the area within the pressure-strain loop.
- Global constructive work (GCW): Defined as positive myocardial work during systolic shortening and negative work of diastolic lengthening during isovolumic relaxation.
- Global wasted work (GWW): Refers to negative myocardial work during lengthening of the left ventricle in systole and shortening during isovolumic relaxation.
- Global work efficiency (GWE): Expressed as a percentage, GWE is calculated by dividing GCW by the sum of GCW and GWW.

Each parameter was reported as a global value, representing the average measurements of all 17 segments. GCW, GWI and GWW are reported as mmHg%. All myocardial work indices were assessed using blinded analysis. Observers were provided with anonymized echocardiographic data to ensure that no clinical or outcome information influenced the assessment.

**Figure 1:**
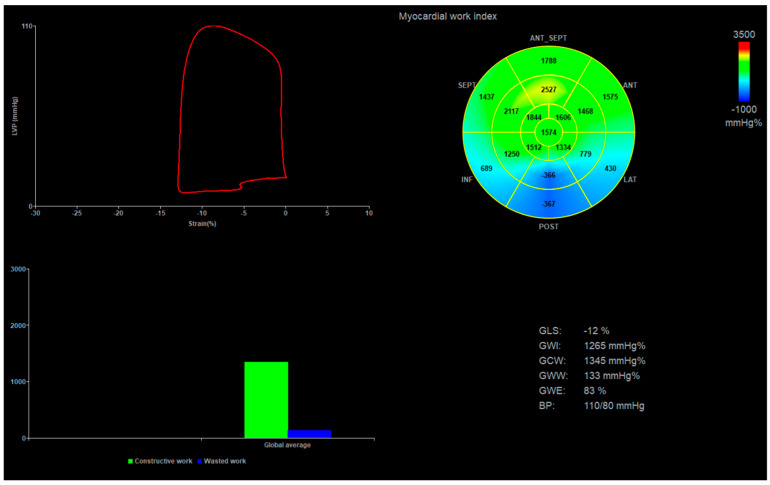
Graphic illustration of myocardial work analysis in a patient with acute inferior ST-elevation myocardial infarction. In the myocardial work Bull’s eye display, green color indicates normal values, while blue highlights areas of negative work. LVP, left ventricular pressure; ANT_SEPT, antero-septal; ANT, anterior; LAT, lateral; POST, posterior; INF, inferior; SEPT, septal; GLS, global longitudinal strain; GWI, global work index; GCW, global constructive work; GWW, global wasted work; GWE, global work efficiency; BP, blood pressure.

Figure 1 illustrates MW measurements in a patient with acute inferior ST-elevation myocardial infarction.

### 2.4. Statistical Analysis

The normality of continuous data was assessed using the Shapiro–Wilk test. Categorical variables are presented as numbers and percentages. Continuous data are expressed as either mean ± standard deviation or median (interquartile range), depending on the distribution of the variables. The Chi-square or Fischer exact test was used to compare categorical variables, as appropriate. For normally distributed continuous data, one-way ANOVA was employed, whereas Kruskal—Wallis’s test was used for continuous variables that did not meet the normality assumptions. Pearson’s or Spearman’s correlation coefficients were used depending on the distribution of the variables, to analyze the correlation between two continuous variables. To assess the diagnostic performance of MW parameters in identifying major adverse outcomes, receiver operating characteristic analysis (ROC) was conducted, with optimal cut-off values determined using the Youden method. Survival analysis was performed using the Kaplan–Meier method to estimate event-free survival rates, and differences between groups were assessed using the log-rank test. Intra- and interobserver variability in MW parameters measurements were assessed using the intraclass correlation coefficient (ICC), based on data from nineteen randomly selected patients evaluated by two independent, blinded observers. Statistical analyses were performed using IBM SPSS software (version 29.0.2.0) and MedCalc (version 22.021). All tests were two-sided, with a *p*-value < 0.05 considered statistically significant.

## 3. Results

### 3.1. Subjects’ Clinical Characteristics

A total of 130 STEMI patients treated with primary PCI, who underwent a complete transthoracic echocardiography prior to discharge, were enrolled in this study. Eleven patients were excluded for the following reasons: the presence of atrial fibrillation at the time of the echocardiographic exam (three patients), severe valvopathy (two patients), inadequate image quality (three patients) and loss to follow-up (three patients). The remaining 119 patients (78.2% man, mean age 58 ± 11 years) form the cohort of our study. During a median follow-up period of 18 ± 13 months, 27 (22.7%) patients had a major adverse event (MAE). Patient’s clinical data and information on treatment at discharge, are summarized in Table 1.

The prevalence of cardiovascular risk factors was high among all included patients, with dyslipidemia being the most common risk factor, affecting 95% of the patients. Killip class I and II were the presenting forms of 97.5% of the patients. A single-vessel disease was identified in 46.2% of patients. The anterior descending artery was the culprit vessel in most cases (43.7) and was the most frequently involved coronary artery among patients with reduced LVEF (62.2%,). In contrast, the right coronary artery was more commonly affected in the group with preserved LVEF (57.6%). Patients with mildly reduced and preserved EF were more likely in Killip Class I at admission (92.7% and 93.9%), while Killip Class II was more frequently observed in those with reduced EF (28.9%). Mineralocorticoid receptor antagonists, loop diuretics, and SGLT2 inhibitors were more frequently administered to patients with reduced EF, while angiotensin-converting enzyme inhibitors and angiotensin II receptor blockers were more frequently prescribed in the group of patients with mildly reduced and preserved LVEF. In alignment with our institution’s treatment protocol and relevant clinical guidelines [13], neprilysin inhibitors (ARNIs) were not included in the management of patients during hospitalization at the time of enrollment in this study. No significant differences were observed across the three LVEF categories in terms of age, gender, heart rate and blood pressure at admission, body mass index, or body surface area.

Among the biological data, the peak values of creatine-kinase isoform MB, Troponin I, alanine aminotransferase and aspartate aminotransferase were significantly higher in patients with STEMI and non-preserved LVEF (Table 2).

Regarding conventional and TDI echocardiographic parameters, LV end-diastolic volume, LV end-systolic volume, LVEF, peak early diastolic mitral annulus velocity (e’), and peak systolic mitral annulus velocity (s’) were statistically significant among the three categories of patients. No differences were found in left atrial volume index, diastolic interventricular septal thickness, LV diastolic posterior wall thickness, peak early diastolic mitral flow velocity (E), peak late transmitral flow velocity (A), E/A or E/e’ ratio. GLS was notably lower in the group with reduced LVEF (−10.4% vs. −13.3% and −15.3%, *p* <0.001). Regarding MW analysis, all MW parameters showed significant different values across the groups (Table 3).

### 3.2. Myocardial Work Analysis Among Different Ejection Fraction Categories

**Figure 2:**
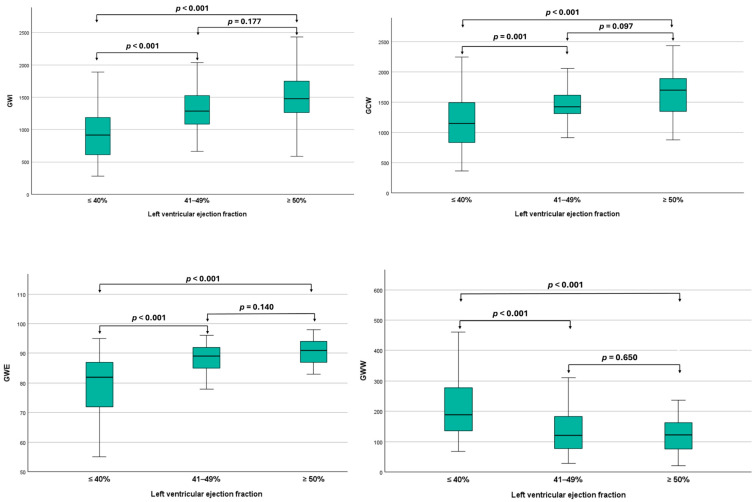
Graphic comparing global work index, global constructive work, global wasted work, and global work efficiency across different left ventricular ejection fraction categories. GWI, global work index; GCW, global constructive work; GWW, global wasted work; GWE, global work efficiency.

A post hoc analysis was performed to compare the mean differences in MW indices across LVEF categories in STEMI patients. Statistically significant differences in all MW parameters were observed between patients with reduced and preserved LVEF, as well as between those with reduced and mildly reduced LVEF. However, no significant differences in MW indices were noted between patients with mildly reduced and preserved LVEF (Figure 2).

### 3.3. Relationship Between Myocardial Work Indices and Left Ventricular Ejection Fraction

**Figure 3:**
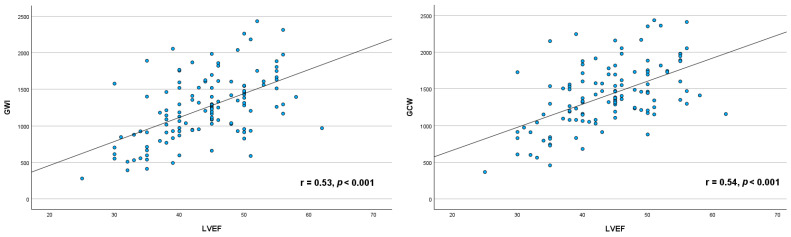

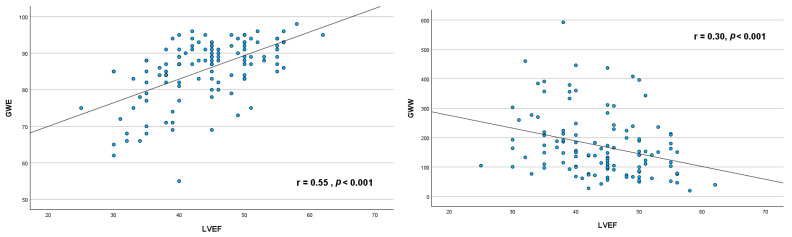
Graphic illustrating the relationship between myocardial work parameters and left ventricular ejection fraction. GWI, global work index; GCW, global constructive work; GWE, global work efficiency; GWW, global wasted work; GLS, global longitudinal strain, LVEF, left ventricular ejection fraction.

Significant moderate positive correlation was found between GWI (r = 0.53, *p* <0.001), GCW (r = 0.54, *p* < 0.001), GWE (r = 0.55, *p* <0.001) and LVEF, whereas weak negative correlation was observed between GWW and LVEF (r = −0.30, *p* < 0.001) (Figure 3).

### 3.4. ROC Analysis in Predicting Major Adverse Events in Acute STEMI Patients

ROC analysis was performed to evaluate the accuracy of s’, E/e’ ratio, GLS, MD and MW indices, to predict MAE in patients with STEMI (Table 4). In the group of patients with preserved LVEF, the highest area under the curve (AUC) was observed for GCW (AUC = 0.730, 95% CI: 0.516–0.944, *p* = 0.035). In the same group, GWI showed borderline statistical significance (AUC = 0.725, 95% CI: 0.491–0.959; *p* = 0.060). No parameters reached statistical significance for predicting MAE in patients with STEMI and mildly reduced EF. Among patients with STEMI and reduced LVEF, GWW had the strongest predictive value (AUC = 0.787, 95% CI: 0.618–0.956, *p* = 0.001), followed by MD (AUC = 0.725, 95% CI: 0.518–0.933, *p* = 0.033).

**Figure 4:**
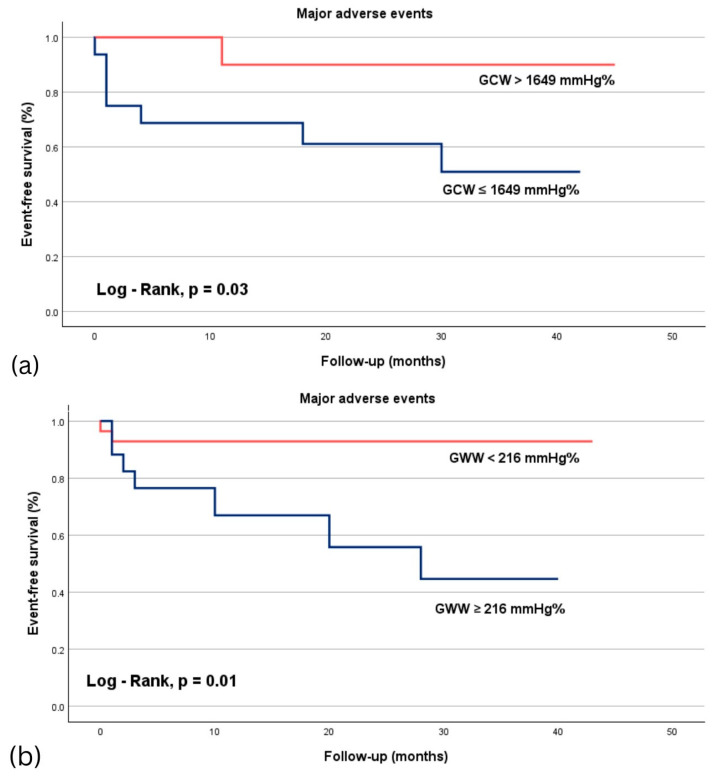
Kaplan–Meier survival curves for major adverse events in STEMI patients, stratified according to the cutoff values obtained by ROC analysis. Panel (**a**) illustrates improved survival rates in patients with preserved LVEF and GCW > 1649 mmHg% (red line), compared to those with GCW ≤ 1649 mmHg% (blue line). Panel (**b**) shows improved event-free survival in patients with reduced LVEF and GWW < 216 mmHg% (red line), compared to those with GWW ≥ 216 mmHg% (blue line). GCW, global constructive work; GWW, global wasted work, LVEF, left ventricular ejection fraction.

In patients with preserved LVEF, the optimal cutoff value for GCW was 1649 mmHg%, with a sensitivity of 87% and a specificity of 64%. For patients with reduced LVEF, the optimal cutoff value for GWW was 216 mmHg%, showing a sensitivity of 77% and a specificity of 73%. Kaplan–Meier curves for MAE in patients with STEMI revealed that those with preserved LVEF and GCW > 1649 mmHg%, had improved survival rates (Log-rank test, *p* = 0.03), while patients with reduced LVEF and GWW < 216 mmHg demonstrate better survival outcomes (Log-rank test, *p* = 0.01) (Figure 4).

### 3.5. The Reliability of MW Parameters

Intra-observer variability was assessed by having the same observer perform measurements at two separate time points, while interobserver variability was evaluated by comparing measurements conducted by two different observers for the same patient. All MW parameters demonstrated high intra-observer agreements, with intraclass correlation coefficients of 0.990 (95% CI: 0.975–0.996) for GWI, 0.990 (95% CI: 0.973–0.996) for GCW, 0.975 (95% CI: 0.935–0.990) for GWW and 0.981 (95% CI: 0.951–0.993) for GWE. Similarly, interobserver variability showed good agreement across all MW indices, with values of 0.970 (95% CI: 0.925–0.988) for GWI, 0.967 (95% CI: 0.918–0.987) for GCW, 0.0.954 (95% CI: 0.885–0.982) for GWW and 0.0.965 (95% CI: 0.911–0.986) for GWE.

## 4. Discussion

In this study, we sought to evaluate the prognostic value of MW indices in predicting outcomes in patients with STEMI treated with primary percutaneous coronary intervention. To our knowledge, this is the first comprehensive analysis of MW indices across three distinct categories of LVEF: reduced (≤40%), mildly reduced (41–49%), and preserved (≥50%). Our preliminary results underscore the added value of MW parameters in enhancing the assessment of myocardial function and improving risk stratification in STEMI patients. These findings highlight the potential of MW measurements as critical tools for refining clinical decision-making and optimizing patient management in this high-risk population.

### 4.1. Differences in MW Indices Across LVEF Categories

Significant differences in MW indices were observed between patients with reduced and preserved LVEF. Severe systolic dysfunction, leading to elevated LV filling pressures and pulmonary congestion, accounts for the higher prevalence of Killip class II and the frequent use of medications targeting fluid overload in patients with an EF ≤ 40%. Anterior STEMI localization involves the left anterior descending artery, which supplies a substantial portion of the myocardium. This results in significant loss of contractility and reduced EF [20], explaining its more frequent association with an EF < 50%. STEMI patients with reduced LVEF (≤40%) exhibited lower global work index (GWI), global constructive work (GCW), and global work efficiency (GWE), alongside higher global wasted work (GWW). These findings reflect impaired myocardial contractility and efficiency in this subgroup. Furthermore, differences in MW indices between reduced and mildly reduced LVEF categories suggest that even modest improvements in LVEF (≤40% to 41–49%) are associated with better myocardial work capacity. However, no significant differences in MW indices were noted between patients with mildly reduced and preserved LVEF. This may be attributed to compensatory mechanisms, such as increased myocardial contractility, or the heterogeneity within the mildly reduced LVEF group, encompassing patients at various stages of recovery or decline.

### 4.2. Correlation Between LVEF and MW Indices

Moderate positive correlations were identified between LVEF and GWI, GCW, and GWE, while weak negative correlations were noted with GWW. These findings suggest that higher LVEF is associated with more effective and efficient myocardial performance, characterized by increased constructive work and reduced wasted work. This relationship aligns with prior studies in healthy individuals [21] and patients with coronary artery disease [22], although differences in study populations may account for variations in correlation strength.

### 4.3. Prognostic Value of MW Indices

ROC analysis revealed the prognostic utility of MW indices in STEMI patients. This suggests that MW can detect nuanced changes in myocardial performance not fully captured by LVEF alone. In the preserved LVEF group, GCW demonstrated the highest predictive ability for MAE (AUC = 0.730, sensitivity 87%, specificity 64%, *p* = 0.035). This underscores GCW’s potential to detect subclinical myocardial dysfunction that may not be evident through LVEF alone. GCW has been shown to predict LVEF reduction in patients with heart failure and preserved LVEF, potentially allowing for earlier identification of those at risk of progressing to reduced LVEF [23]. Conversely, in the reduced LVEF group, GWW exhibited the strongest predictive value (AUC = 0.787, *p* = 0.001), highlighting the importance of assessing not just the amount of work but also the efficiency and homogeneity of myocardial contractions. Parameters such as mechanical dispersion (MD) also demonstrated prognostic relevance, reinforcing the importance of assessing mechanical heterogeneity and ventricular instability.

### 4.4. Pathophysiological Implications

Elevated GWW reflects energy expanded in ineffective contractions, often driven by myocardial desynchrony, ischemia, or fibrosis [24,25,26]. GWW may reveal negative work through two mechanisms: systolic lengthening and/or postsystolic shortening. The greater the amplitude and duration of systolic lengthening, the lower the likelihood that a myocardial segment is viable. A segment exhibiting postsystolic shortening may either be non-viable, behaving passively like an elastic recoil after the aortic valve closure, or it may be a viable but compromised segment, that continues to shorten weakly and slowly after aortic valve closure, when LV pressure falls [27]. This highlights the value of GWW in assessing the prognosis of patients with myocardial damage and impaired contractile function [28]. Lustosa et al. [29] reported that GWW was the only MW parameter that remained unchanged three months after STEMI, suggesting its potential role as a marker of myocardial scar tissue. The heterogeneity of scar tissue is known to contribute to slow conduction, which can create a substrate for severe arrhythmias following STEMI [30]. Similarly, increased MD, indicative of inhomogeneous contraction patterns, contributes to myocardial inefficiency and remodeling [31,32,33]. Increased MD results in abnormal contraction patterns and myocardial stretch, which are likely to elevate myocardial wasted work and reduce myocardial efficiency [34].

### 4.5. Clinical Implications

MW analysis provides additional insights into myocardial function beyond traditional LVEF assessment. GCW’s predictive value in preserved LVEF patients suggests its utility in early identification of subclinical dysfunction, enabling timely therapeutic interventions. In reduced LVEF patients, GWW’s strong association with adverse outcomes underscores the importance of addressing myocardial inefficiency through tailored treatment strategies. By providing a more nuanced assessment of myocardial function than traditional measures like LVEF and GLS, MW indices could aid in risk stratification and therapy optimization. Our results underscore the potential utility of MW as prognostic tools in the management of STEMI patients. For instance, patients with impaired MW parameters might benefit from more intensive monitoring or tailored therapeutic strategies to improve outcomes. Future research should focus on integrating MW into clinical decision-making pathways to validate its role in guiding treatment in acute ischemic settings. Another important aspect for future studies is the inclusion of echocardiographic assessments beyond the initial hospitalization. Given the critical role of LV remodeling, a well-established factor influencing post-STEMI outcomes, serial imaging is essential to understand the dynamic changes in LV structure over time and their association with long-term outcomes in this patient population.

### 4.6. Study Limitations

Our study is subject to several limitations. Firstly, its single-center design and the relatively short follow-up period may constrain the robustness of our conclusions. Secondly, the potential impact of interobserver variability in MW measurements should be considered. The small sample size could also affect the statistical power needed to detect significant differences in subgroup analyses, thereby limiting the generalizability of our results. Moreover, by excluding non-STEMI patients and those with significant R-R variability on electrocardiogram (such as individuals with atrial fibrillation, frequent supraventricular or ventricular ectopic beats, etc.), the study’s findings may not be applicable to a broader patient population. Notably, no conventional echocardiographic or MW parameter proved statistically significant in predicting MAE among patients with STEMI and mildly reduced LVEF. To enhance the robustness and applicability of MW indices in the management of STEMI, future research should involve multicenter studies with extended follow-up periods.

## 5. Conclusions

This study highlights the prognostic utility of MW indices, specifically GCW and GWW, in STEMI patients across various LVEF categories. Notably, GCW was identified as a strong predictor of adverse outcomes in patients with preserved LVEF, whereas GWW showed robust predictive capability in those with reduced LVEF. These results highlight the added benefit of MW analysis beyond conventional LVEF evaluations, presenting new avenues for enhanced risk stratification and tailored management strategies in this vulnerable group.

## Figures and Tables

**Table 1:** Patient’s characteristics and treatment at discharge.

Variable	All Patients (*n* = 119)	STEMI with EF ≥50% (*n* = 33)	STEMI with EF 41–49% (*n* = 41)	STEMI with EF ≤40% (*n* = 45)	*p* Value
Clinical characteristics					
Man, n (%)	93 (78.2)	28 (84.8)	33 (80.5)	32 (71.1)	0.326
Age, years	58 ± 11	54 ± 9	57 ± 12	60 ± 11	0.339
BMI, kg/m^2^	27.4 (24.9–31.06)	27.6 (25.9–30.6)	28.4 (25.9–31.2)	27.4 (25.1–32.1)	0.979
BSA, m^2^	2.0 ± 0.2	1.9 ± 0.1	2 ± 0.2	1.9 ± 0.2	0.755
SBP, mmHg	146 ± 24	148 ± 28	145 ± 22	145 ± 22	0.734
DBP, mmHg	88 ± 15	89 ± 14	90 ± 16	86 ± 14	0.837
Heart rate, bpm	79 ± 14	77 ± 11	78 ± 12	82 ± 17	0.095
Cardiovascular risk factors					
Dyslipidemia, n (%)	113 (95.0)	32 (97.0)	39 (95.1)	42 (93.3)	0.877
Hypertension, n (%)	90 (75.6)	22 (66.7)	30 (73.2)	38 (84.4)	0.176
Smoking, n (%)	79 (66.4)	24 (72.7)	27 (65.9)	28 (62.2)	0.622
Diabetes, n (%)	24 (20.2)	6 (18.2)	7 (17.1)	11 (24.4)	0.658
Cardiac inheritance, n (%)	8 (6.7)	2 (6.1)	1 (2.4)	5 (11.1)	0.340
Previous CAD, n (%)	4 (3.4)	0 (0.0)	3 (7.3)	1 (2.2)	0.316
Killip class at admission					
Class I, n (%)	99 (83.2)	31 (93.9)	38 (92.7)	30 (66.7)	<0.001
Class II, n (%)	17 (14.3)	1 (3.0)	3 (7.3)	13 (28.9)	0.003
Class III, n (%)	2 (1.7)	1 (3.0)	0 (0.0)	1 (2.2)	0.735
Class IV, n (%)	1 (0.8)	0 (0.0)	0 (0.0)	1 (2.2)	1.000
Culprit vessel					
Left main coronary artery, n (%)	1 (0.8)	0 (0)	1 (2.4)	0 (0)	0.383
Left anterior descending artery, n (%)	52 (43.7)	7 (21.2)	17 (41.5)	28 (62.2)	0.001
Circumflex coronary artery, n (%)	22 (18.5)	7 (21.2)	9 (22.0)	6 (13.3)	0.527
Right coronary artery, n (%)	44 (37.0)	19 (57.6)	14 (34.1)	11 (24.4)	0.010
Number of affected vessels					
Single-vessel disease, n (%)	55 (46.2)	20 (60.6)	15 (36.6)	20 (44.4)	0.114
Two-vessel disease, n (%)	40 (33.6)	7 (21.2)	18 (43.9)	15 (33.3)	0.121
Three-vessel disease, n (%)	24 (20.2)	6 (18.2)	8 (19.5)	10 (22.2)	0.900
Treatment at discharge					
Aspirin, n (%)	120 (99.2)	33 (100)	41 (100)	44 (97.8)	1.00
P_2_Y_12_i, n (%)	121 (100)	34 (100)	42 (100)	45 (100)	
Statins, n (%)	121 (100)	30 (100)	36 (100)	37 (100)	
ACE inhibitor/ARBs n (%)	79 (66.4)	24 (72.7)	32 (78.0)	23 (51.1)	0.020
Nitrate, n (%)	54 (45.4)	12 (36.4)	22 (53.7)	20 (44.4)	0.328
Betablocker, n (%)	87 (73.1)	22 (66.7)	30 (73.2)	35 (77.8)	0.550
Loop diuretics, n (%)	90 (75.6)	17 (51.5)	32 (78.0)	41 (91.1)	<0.001
MRAs, n (%)	93 (78.2)	19 (57.6)	32 (78.0)	42 (93.3)	0.001
SGLT2i, n (%)	19 (16.0)	1 (3.0)	2 (4.9)	16 (35.6)	<0.001

Values are reported as mean ± standard deviation or median (interquartile range). STEMI, ST-elevation myocardial infarction; EF, ejection fraction; BMI, body mass index; BSA, body surface area; SBP, systolic blood pressure; DBP, diastolic blood pressure; CAD, coronary artery disease; P_2_Y_12_i, inhibitors of P_2_Y_12_ receptors; ACE, angiotensin-converting enzyme; ARBs, angiotensin II receptor blockers; MRAs, mineralocorticoid receptor antagonists; SGLT2i, sodium-glucose co-transporter 2 inhibitor.

**Table 2:** Biological variables and their association with left ventricular ejection fraction categories in STEMI patients.

Variable	All Patients (*n* = 119)	STEMI with EF ≥50% (*n* = 33)	STEMI with EF 41–49% (*n* = 41)	STEMI with EF ≤40% (*n* = 45)	*p* Value
Glycemia, mg/dL	126 (108–152)	114 (103–135)	123 (108–144)	148 (110–187)	0.064
Total cholesterol, mg/dL	189 ± 42	190 ± 40	191 ± 47	185 ± 40	0.940
LDLc, mg/dL	118 ± 29	120.9 ± 31.3	119.0 ± 27.1	116.1 ± 30.1	0.875
Triglycerides, mg/dL	134 (95–169)	151 (97–200)	143 (95–172)	120 (78–151)	0.156
Creatinine level, mg/dL	0.95 (0.83–1.13)	0.90 (0.82–1.04)	0.92 (0.77–1.10)	0.95 (0.84–1.17)	0.699
eGFR, ml/min/1.73 m^2^	85.0 ± 21.6	88.8 ± 18.9	87.9 ± 21.4	79.3 ± 23.2	0.468
Hemoglobin, g/dL	14.6 ± 1.5	14.6 ± 1.5	14.5 ± 1.5	14.5 ± 1.4	0.976
Leukocytes, 10^3^/µL	12.0 ± 3.3	11.4 ± 3.5	12.2 ± 3.5	12.2 ± 3.1	0.243
Neutrophiles, 10^3^/µL	7.44 (6.47–8.17)	7.44 (6.57–8.15)	7.52 (6.80–8.18)	7.97 (7.24–8.52)	0.377
ESR, mm/h	12 (6–18)	10 (6–14)	10 (6–15)	10 (5–20)	0.560
Troponin I, ng/L *	6849 (333–34,857)	1405 (188–13,339)	9337 (1182–40,000)	6942 (262–39,356)	0.026
CK-MB, U/L *	221 (95.5–379)	181 (61–270)	249 (118–359)	259 (115–483)	0.031
ALT, U/L *	59 (45–90)	50 (36–71)	57 (47–81)	82 (49–103)	0.020
AST, U/L *	229 (113–378)	182 (65–276)	217 (95–313)	312 (173–566)	0.003

Values are expressed as mean ± standard deviation or median (interquartile range). * The peak values during the index admission. ALT, alanine aminotransferase; AST, aspartate aminotransferase; CK-MB, creatine-kinase isoform MB; eGFR, estimated glomerular filtration rate; ESR, erythrocyte sedimentation rate; LDLc, low-density lipoprotein cholesterol; STEMI, acute ST-segment elevation myocardial infarction; EF, ejection fraction.

**Table 3:** Echocardiographic parameters and their association with left ventricular ejection fraction categories in STEMI patients.

Variable	All Patients (*n* = 119)	STEMI with EF ≥ 50% (*n* = 33)	STEMI with EF 41–49% (*n* = 41)	STEMI with EF ≤ 40% (*n* = 45)	*p* Value
LA volume index (mL/m^2^)	31.2 ± 10.9	30.4 ± 9.1	31.2 ± 10.0	31.8 ± 12.9	0.827
LVEDV (mL)	109.4 ± 26.1	101 ± 25	111 ± 29	114 ± 23	0.014
LVESV (mL)	62 (45–72)	45 (41–61)	60 (47–70)	68 (54–77)	<0.001
LVEF (%)	44 ± 7	53 ± 3	44 ± 2	36 ± 3	<0.001
IVS (cm)	1.21 ± 0.2	1.14 ± 0.2	1.25 ± 0.16	1.23 ± 0.17	0.254
PWT (cm)	1.15 ± 0.1	1.11 ± 0.1	1.16 ± 0.1	1.18 ± 0.1	0.271
E wave (m/s)	0.65 ± 0.1	0.65 ± 0.2	0.68 ± 0.1	0.64 ± 0.1	0.607
A wave (m/s)	0.75 ± 0.1	0.75 ± 0.1	0.76 ± 0.2	0.76 ± 0.2	0.795
E/A ratio	0.8 (0.6–1.1)	0.7 (0.5–1.1)	0.8 (0.7–1.1)	0.7 (0.6–1.2)	0.544
e’ wave (m/s)	0.06 ± 0.02	0.07 ± 0.02	0.06 ± 0.01	0.05 ± 0.01	<0.001
s’ wave (m/s)	0.07 ± 0.01	0.08 ± 0.02	0.07 ± 0.01	0.06 ± 0.01	<0.001
E/e’ ratio	10 (7.8–12.2)	9.2 (6.8–10.9)	10 (8.1–12.5)	10 (8.1–15.0)	0.111
LV global longitudinal strain (%)	−12.8 ± 3.4	−15.3 ± 2.9	−13.3 ± 2.3	−10.4 ± 3.1	<0.001
LV mechanical dispersion (ms)	74.0 ± 24.4	65.8 ± 22.5	74.2 ± 22.0	80.4 ± 26.6	0.061
GWI, mmHg%	1276 ± 431	1540 ± 414	1298 ± 308	1048 ± 428	<0.001
GCW, mmHg%	1445 ± 412	1693 ± 386	1454 ± 292	1243 ± 4229	<0.001
GWW, mmHg%	148 (95–213)	123 (71–158)	133 (74–199)	191 (143–305)	<0.001
GWE, %	88 (82–92)	92 (87–94)	89 (84–92)	84 (73–87)	<0.001

Values are expressed as mean ± standard deviation or median (interquartile range). STEMI, ST elevation myocardial infarction; EF, ejection fraction; LA, left atrium; LVEDV, left ventricular end-diastolic volume; LVESV, left ventricle end-systolic volume; LVEF, left ventricular ejection fraction; IVS, diastolic interventricular septal thickness; PWT, diastolic posterior wall thickness; E, peak early diastolic mitral flow velocity; A, peak late transmitral flow velocity; e’, peak early diastolic mitral annulus velocity; s’, peak systolic mitral annulus velocity; LV, left ventricle; GWI, global work index; GCW, global constructive work; GWW, global wasted work; GWE, global work efficiency.

**Table 4:** Receiver operating characteristic analysis for predicting major adverse events in STEMI patients across various LVEF categories.

Parameters	AUC (95% CI)	*p* Value
Preserved LVEF (≥50%)		
s’ wave	0.640 (0.425–0.855)	0.361
E/e’ ratio	0.378 (0.170–0.585)	0.248
Global longitudinal strain	0.643 (0.384–0.901)	0.280
Mechanical dispersion	0.650 (0.419–0.881)	0.203
GWI	0.725 (0.491–0.959)	0.060
GCW	0.730 (0.516–0.944)	0.035
GWW	0.533 (0.292–0.773)	0.791
GWE	0.625 (0.385–0.8765)	0.307
Mildly reduced LVEF (41–49%)		
s’ wave	0.415 (0.228–0.601)	0.368
E/e’ ratio	0.406 (0.212–0.600)	0.447
Global longitudinal strain	0.531 (0.321–0.740)	0.775
Mechanical dispersion	0.571 (0.355–0.787)	0.519
GWI	0.616 (0.398–0.835)	0.298
GCW	0.645 (0.429–0.862)	0.189
GWW	0.482 (0.270–0.695)	0.870
GWE	0.561 (0.353–0.770)	0.564
Reduced LVEF (≤40%)		
s’ wave	0.469 (0.278–0.660)	0.756
E/e’ ratio	0.520 (0.324–0.716)	0.841
Global longitudinal strain	0.571 (0.353–0.789)	0.524
Mechanical dispersion	0.725 (0.518–0.933)	0.033
GWI	0.519 (0.287–0.751)	0.876
GCW	0.451 (0.234–0.667)	0.667
GWW	0.787 (0.618–0.956)	0.001
GWE	0.657 (0.445–0.870)	0.147

s’, peak systolic mitral annulus velocity; E, peak early diastolic mitral flow velocity; e’, peak early diastolic mitral annulus velocity; GWI, global work index; GCW, global constructive work; GWW, global wasted work; GWE, global work efficiency; AUC, area under the curve; LVEF, left ventricular ejection fraction.

## Data Availability

The data underlying this article is kept confidential to protect participant privacy. However, interested parties may request access by contacting the correspondent author.

## Abbreviations

The following abbreviations are used in this manuscript:

A	Peak late transmitral flow velocity
AUC	Area Under the Curve
CI	Confidence Interval
e’	Peak early diastolic mitral annulus velocity
E	Peak early diastolic mitral flow velocity
GCW	Global Constructive Work
GLS	Global Longitudinal Strain
GWE	Global Work Efficiency
GWI	Global Work Index
GWW	Global Wasted work
LV	Left ventricle
MAE	Major Adverse Event
MW	Myocardial Work
PCI	Percutaneous Coronary Intervention
s’	Peak systolic mitral annulus velocity
STEMI	ST-elevation myocardial infarction
